# High coronal alignment accuracy and satisfactory early outcomes using augmented reality assisted kinematic alignment in total knee arthroplasty

**DOI:** 10.1002/jeo2.70476

**Published:** 2025-10-28

**Authors:** Giorgio Cacciola, Francesco Bosco, Daniele Vezza, Matteo Schirò, Francesco Carturan, Gianpaolo Gazziero, Marco Bufalo, Luigi Sabatini

**Affiliations:** ^1^ Department of Orthopaedics and Traumatology University of Turin Turin Italy; ^2^ Department of Robotic and Mini‐Invasive Orthopaedic Surgery Humanitas “Gradenigo” Hospital Turin Italy; ^3^ Department of Precision Medicine in Medical, Surgical and Critical Care (Me.Pre.C.C.) University of Palermo Palermo Italy; ^4^ Department of Orthopaedics and Traumatology G.F. Ingrassia Hospital Unit Palermo Italy

**Keywords:** augmented reality, component positioning, computer‐assisted surgery, kinematic alignment, outcomes, surgical precision, total knee arthroplasty

## Abstract

**Purpose:**

Accurate component positioning in total knee arthroplasty (TKA) is critical for implant longevity and patient satisfaction. Augmented reality (AR)‐based navigation systems offer enhanced precision and intraoperative versatility. This study evaluated the accuracy of component positioning, implant sizing and short‐term clinical outcomes of a novel AR‐assisted navigation system (NextAR, Medacta International) in TKA using a modified kinematic alignment (KA) technique.

**Methods:**

Forty‐one consecutive patients underwent primary TKA using AR‐assisted navigation with ≥12‐month follow‐up. Preoperative CT‐based 3D planning optimised cut orientation and component placement. All received a cemented medial pivot prosthesis (GMK Sphere) with full femoral resurfacing following a KA protocol. Tibial cuts were guided intraoperatively by real‐time ligament balancing. Planned versus achieved positions were compared on radiographs. Western Ontario and McMaster Universities Osteoarthritis Index (WOMAC), forgotten joint score (FJS) and range of motion (ROM) were recorded pre‐ and postoperatively and analysed using paired *t*‐tests (*p* < 0.05).

**Results:**

The average difference between planned and postoperative alignment was 0.05° ± 0.76° for LDFA, 0.1° ± 0.6° for MPTA, –0.5 ± 1.7° for femoral component flexion, and 0.3° ± 1.3° for PTS. Root mean square errors were 0.75°, 1.23°, 1.73° and 1.34°, respectively. Postoperative HKA improved from 174.3° ± 3.4° to 177.8° ± 2.1° (*p* < 0.001). Component size prediction was accurate in 100% of femurs and 95.1% of tibias. At final follow‐up (14.2 ± 2.3 months), WOMAC improved from 51.5 ± 16.7 to 13.6 ± 5.3, FJS from 26.2 ± 9.6 to 82.2 ± 7.4, flexion from 103.3° ± 17.4° to 129.4° ± 7.2° and extension from 3.3° ± 0.43° to 0.1° ± 0.28° (all *p* < 0.001).

**Conclusions:**

AR‐based navigation in modified KA‐TKA ensured accurate LDFA restoration and femoral sizing, with good short‐term outcomes. Variability remained in MPTA, femoral flexion and PTS. Although no coronal recuts were needed, two tibial recuts for tight extension gaps highlight areas for system refinement.

**Level of Evidence:**

Level IV.

**Abbreviations**
3D: three‐dimensional
aHKA: anatomic hip‐knee‐ankle angle
AR: augmented reality
BMI: body mass index
CAS: computer‐assisted surgery
CPAK: coronal plane alignment of the knee
CT: computed tomography
FJS: forgotten joint score
FMA: femoral mechanical axis
HKA: hip‐knee‐ankle angle
KA: kinematic alignment
LCL: lateral collateral ligament
LDFA: lateral distal femoral angle
MA: mechanical alignment
MCL: medial collateral ligament
MPTA: medial proximal tibial angle
PCA: posterior condylar axis
PCL: posterior cruciate ligament
PROMs: patient‐reported outcome measures
PTS: posterior tibial slope
RMS: root mean square
RMSE: root mean square error
ROM: range of motion
SD: standard deviation
TEA: transepicondylar axis
TKA: total knee arthroplasty
WOMAC: Western Ontario and McMaster Universities Osteoarthritis Index

## INTRODUCTION

Precise component positioning in total knee arthroplasty (TKA) is a critical determinant of implant longevity, functional recovery and overall patient satisfaction, regardless of the alignment strategy employed [10, 14, 31, 29].

In mechanically aligned TKA, varus positioning of the tibial component has been associated with an increased risk of aseptic loosening due to asymmetric load distribution and higher stresses in the medial compartment [14, 31]. Conversely, in kinematically aligned (KA) TKA, moderate varus alignment of the tibial component—when restoring the patient's native joint line—is not associated with higher failure rates or worse clinical outcomes [10, 11]. Howell et al. demonstrated that long‐term implant survivorship and function remain favourable even with residual varus in the tibial component, provided that the pre‐arthritic alignment is respected [10].

Rather than focusing on a fixed posterior tibial slope (PTS) threshold, recent evidence emphasises the importance of preserving the patient's native slope. Howell et al. reported that reductions in PTS relative to the contralateral limb were associated with tibial component failure, likely due to posterior shear overload and altered flexion kinematics [10].

The advent of computer‐assisted surgery (CAS) has markedly improved the reproducibility and accuracy of TKA component placement [3, 18, 39]. Among the most recent technological innovations, augmented reality (AR) systems offer intraoperative guidance by projecting virtual axes, angles and planning references directly into the surgeon's field of view via head‐mounted displays or smart glasses [15, 36]. Compared to robotic platforms, AR systems offer a more compact design, lower cost and improved workflow integration [15, 16]. However, the current clinical literature on AR‐assisted TKA remains limited, primarily restricted to preclinical or cadaveric investigations [2, 5, 32, 33, 34, 38].

To address this gap, we recently introduced a TKA technique that combines a modified KA approach with intraoperative soft tissue assessment via an AR‐assisted navigation platform (NextAR, Medacta International) [6, 27]. This modification preserves KA principles for femoral resurfacing. Still, it adapts the tibial cut intraoperatively according to ligament balance, in a manner like the technique described by Howell, with the addition of CT‐based preoperative planning and AR guidance [21, 22].

A known limitation of calipered KA is the assumption of uniform cartilage thickness—commonly 2 mm on the distal and posterior femoral condyles—used during resection planning in combination with the 1 mm saw blade [19]. In clinical practice, cartilage wear is often asymmetric and variable, particularly in advanced osteoarthritis. Giurazza et al. reported that the average cartilage thickness across compartments can vary significantly, ranging from 1.95 to 2.6 mm, depending on the location [8, 9]. As a result, unaccounted discrepancies in cartilage thickness may introduce unintended deviations in alignment. Computed tomography (CT)‐based navigation systems, which rely exclusively on osseous landmarks, can help eliminate this variability and improve resection accuracy [6, 27].

This study aimed to evaluate the radiographic accuracy of an AR‐based navigation system in modified KA‐TKA by comparing planned and postoperative component positioning (lateral distal femoral angle [LDFA], medial proximal tibial angle [MPTA], PTS and femoral flexion), assessing its precision in predicting implant sizes and reporting short‐term clinical outcomes after a minimum 1‐year follow‐up.

We hypothesise that CT‐based AR guidance can accurately replicate the planned bone resections and enable intraoperative adjustments based on soft tissue behaviour, thereby improving the precision of component placement in kinematically aligned TKA.

## MATERIALS AND METHODS

### Study design and ethical approval

Following approval from the institutional Ethics Committee (approval code: Territorial Ethics Committee A.O.U.

Città della Salute e della Scienza di Torino 123/2024), a retrospective observational study was conducted on patients who underwent primary modified KA‐TKA with the support of an AR‐based navigation system (NextAR, Medacta International, Castel San Pietro). All consecutive patients who met the inclusion criteria were enrolled in the study. Written informed consent was obtained from each patient at the time of the 1‐year clinical follow‐up evaluation. No patient refused to provide consent for participation in the study. The same senior surgeon (L.S.) performed all procedures at a single referral centre. In every case, a cemented medial pivot implant (GMK Sphere, Medacta International) was used. The patella was never resurfaced, but in each case, an accurate reshaping and peripheral denervation were performed.

### Inclusion and exclusion criteria

Patients were eligible for inclusion if they met the following criteria: a diagnosis of end‐stage knee osteoarthritis (Kellgren–Lawrence grade III or IV), an age between 40 and 80 years, the availability of high‐quality preoperative and postoperative standing long‐leg radiographs and a minimum of 12 months of clinical and radiographic follow‐up. All the surgeries were performed between March 2023 and March 2024. Patients were excluded if they had secondary osteoarthritis (e.g., posttraumatic or related to systemic inflammatory conditions), a history of previous surgical procedures on the index knee, or neurological disorders potentially affecting gait or limb function.

### Surgical technique

The NextAR system (Medacta International, Castel San Pietro) is a CT‐based navigation platform with AR support, enabling surgeons to perform precise 3D planning, execute accurate bone resections and receive real‐time intraoperative feedback on collateral ligament balance throughout the full range of motion (ROM) [6, 27]. The surgical technique adopted in this study represents a modification of the traditional unrestricted kinematic alignment (uKA) protocol. The femoral component is implanted according to KA principles—resurfacing the distal and posterior condyles without altering the native joint line—while the tibial resection is planned intraoperatively based on the behaviour of the collateral ligaments after femoral resurfacing. This technique represents a modified form of the KA technique. Like Howell's original method [21, 22], the tibial cut is adjusted according to ligament balance after femoral resurfacing; however, our approach integrates CT‐based preoperative planning, AR navigation and real‐time digital ligament tension assessment to refine bone resection parameters. This strategy preserves the KA philosophy but introduces controlled intraoperative adaptability to achieve physiological ligament balance, particularly in cases of asymmetric tibial wear or altered soft tissue tension.

The AR system utilises smart glasses worn by the surgeon, which enable the real‐time projection of planning data and alignment targets directly into the visual field. This allows the surgeon to maintain continuous focus on the operative field. In our series, the glasses were used during the positioning of the femoral cutting guides to streamline workflow and reduce operative time.

#### CT scan acquisition and 3D planning

**Figure 1:**
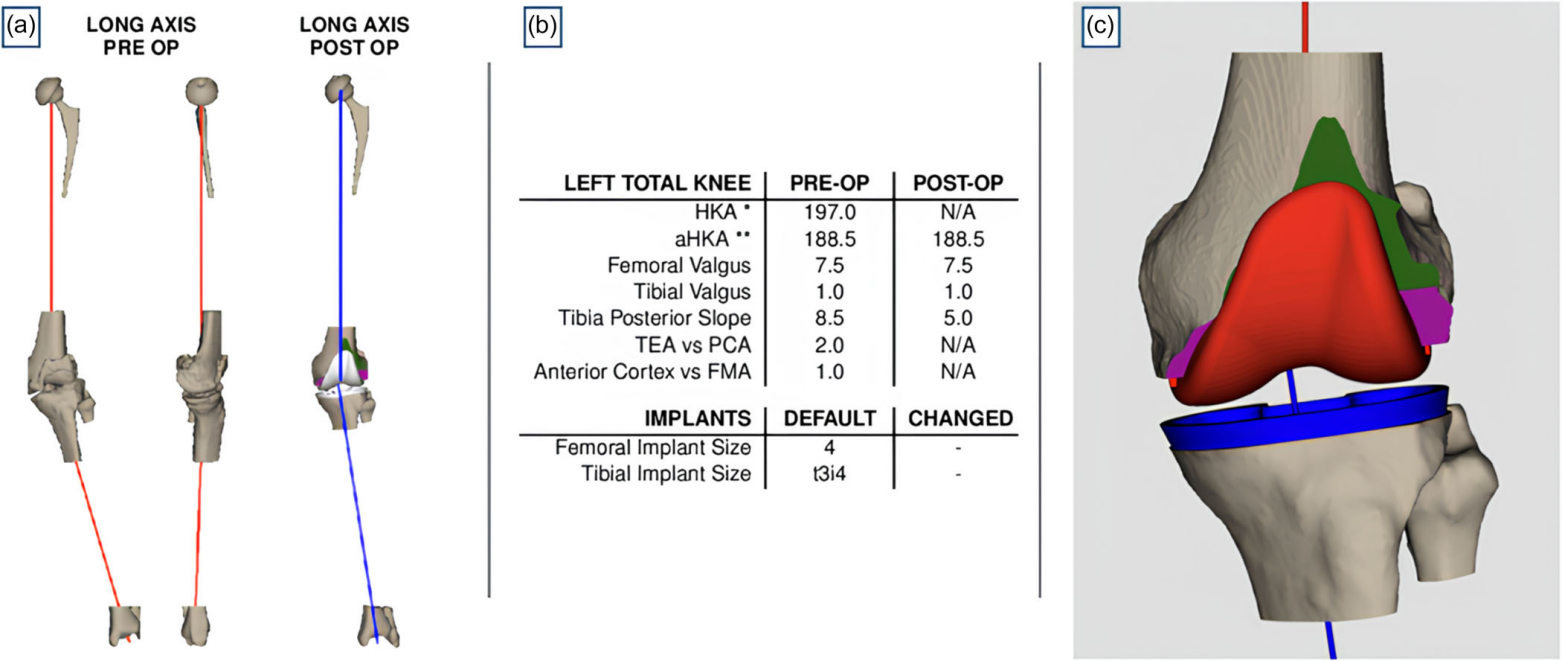
Three‐dimensional preoperative planning. (a) Preoperative assessment of lower limb alignment. The right panel shows coronal plane alignment, the centre panel displays sagittal plane alignment and the left panel illustrates the predicted postoperative alignment based on planned angles (in this case, following an unrestricted kinematic alignment strategy). (b) Summary table reporting preoperative and planned angular measurements, along with the planned sizes of the femoral and tibial components. (c) Detailed visualisation of the final component positioning, including predictions of the planned femoral and tibial resections. aHKA, anatomic hip‐knee‐ankle angle; FMA, femoral mechanical axis; HKA, hip‐knee‐ankle angle; PCA, posterior condylar axis; TEA, transepicondylar axis.

A full‐length CT scan (pelvis, knee and ankle) is obtained to evaluate the coronal and axial alignment of the lower limb. Once uploaded to the manufacturer's cloud platform, the scan is segmented, and a 3D reconstruction of the knee joint is created [6, 27]. The system provides detailed parameters, including the mechanical axis, joint line orientation and rotational references (Figure 1). Femoral planning includes resurfacing the coronal and axial planes and preserving the LDFA and posterior condylar axis (PCA). For femoral component flexion, the system suggests the optimal angle that prevents anterior notching while maximising implant congruity.

Tibial planning initially aims to replicate the patient's native MPTA and PTS; however, the final PTS is intentionally adjusted intraoperatively following femoral resurfacing to account for posterior cruciate ligament (PCL) resection and to achieve balanced flexion‐extension gaps.

Collateral ligament balance is reassessed intraoperatively after femoral resurfacing and placement of the trial femoral component. Based on these findings, the tibial cut is adjusted to optimise soft tissue balance rather than strictly following the preoperative plan [6]. The GMK Sphere prosthesis, combined with the GMK Sphere Flex Tibial Insert (Medacta International), was used in all procedures. This implant features a highly congruent medial ball‐in‐socket articulation, offering intrinsic stability and minimising anteroposterior translation in the medial compartment during flexion and extension. Conversely, the lateral compartment is flat, allowing the femoral condyle to roll back as flexion increases [6, 27]. As the insert design requires PCL resection, a key difference from Howell's original calibrated KA technique [21, 22] is a reduction in PTS to compensate for the increased flexion gap resulting from PCL removal. The final PTS is selected intraoperatively based on soft tissue tension and assessment of the flexion‐extension gap [9, 19].

#### Navigated femoral resurfacing

**Figure 2:**
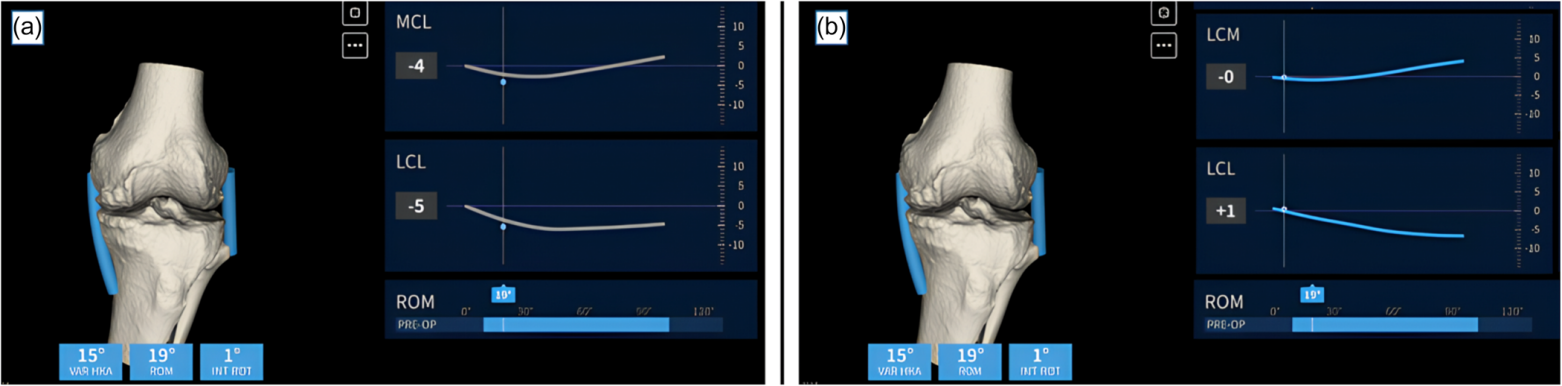
Intraoperative evaluation of collateral ligament behaviour. (a) Initial intraoperative assessment. The left panel displays lower limb alignment (showing a 15° varus deformity), ROM and tibial rotation. The right panel illustrates the behaviour of the MCL and LCL throughout the full ROM. This graph represents a typical case of varus deformity associated with cartilage wear of the medial femoral condyle. Ideally, the MCL should demonstrate symmetric tension throughout motion; however, in this case, the MCL appears shortened in extension, clinically corresponding to medial joint line opening under valgus stress. Conversely, the LCL shows a physiological pattern, with maximum tension in extension that progressively decreases with increasing flexion. (b) Intraoperative assessment after femoral resurfacing with the trial femoral component in place, demonstrating the corrected behaviour of the collateral ligaments after compensating for femoral cartilage loss. LCL, lateral collateral ligament; MCL, medial collateral ligament; ROM, range of motion.

**Figure 3:**
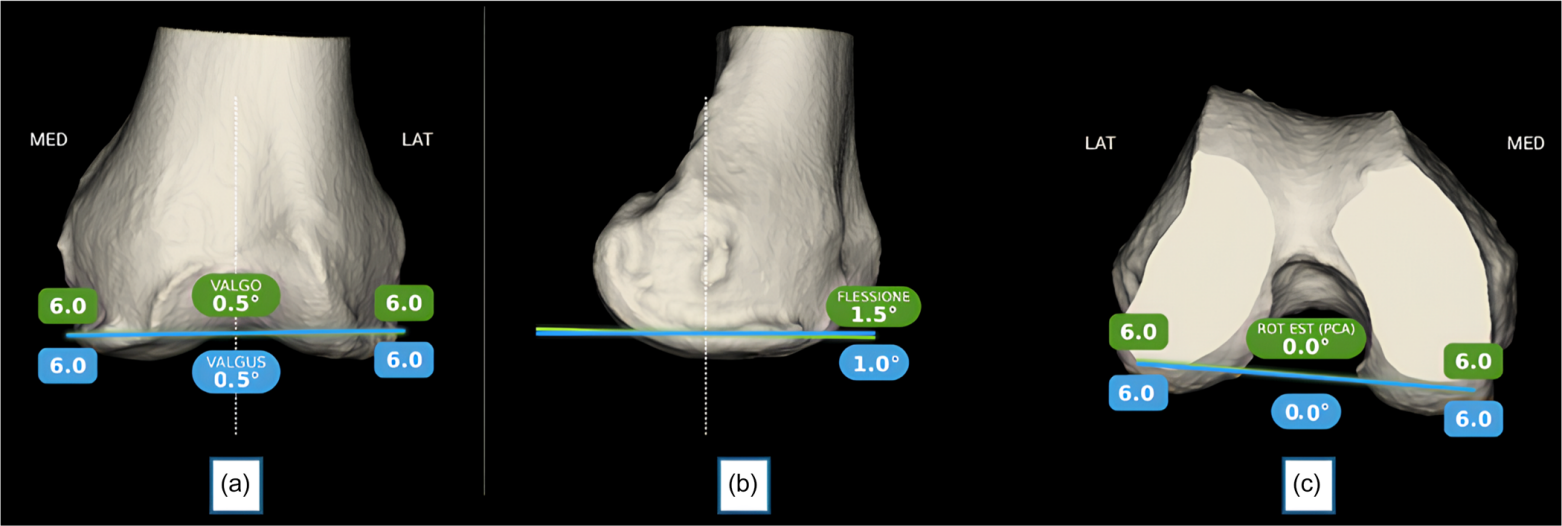
Execution of distal and posterior femoral cuts using augmented reality (AR) navigation. (A) In the coronal plane, the real‐time position of the cutting guide (blue line) is matched with the planned trajectory (green line) to achieve the desired distal femoral resection. (B) In the sagittal plane, the alignment between the real‐time guide position (blue line) and the planned cut (green line) is verified to ensure accurate flexion angle. (C) The posterior femoral cut is executed with 0° rotation relative to the posterior condylar axis (PCA), confirming precise rotational alignment.

Following registration of the femoral surface, an initial assessment of medial collateral ligament (MCL) and lateral collateral ligament (LCL) balance is performed (Figure 2). Using the AR navigation system, the real‐time position of the Cutting Guide (blue line) is matched with the planned trajectory (green line) in both the coronal (Figure 3a) and sagittal planes (Figure 3b). The posterior femoral cut is then executed, oriented at 0° relative to the PCA (Figure 3c). After completing the distal and posterior femoral resections, osteophyte removal from the femur and tibia is performed. This step is postponed until this stage to avoid compromising the CT‐bone surface registration required for accurate navigation. However, osteophyte removal is crucial to ensure a valid intraoperative evaluation of collateral ligament behaviour. Subsequently, a trial femoral component is positioned on the resurfaced femur to verify fit, rotational alignment and coronal positioning.

#### Collateral ligament behaviour and tibial resection planning

Following femoral resurfacing, the pattern of ligament balance is reassessed, and any compensable tibial wear is identified, typically medial in varus knees or lateral in valgus knees. In the absence of tibial wear, the collateral ligaments are expected to follow native behaviour, with the MCL maintaining isometry throughout the arc of motion and the LCL gradually relaxing in flexion. In cases where asymmetric tibial wear is present, the ligament balance curves will deviate from the expected pattern. To restore physiological tension, the tibial resection parameters—including resection depth, varus/valgus angle and PTS—are modified intraoperatively until targeted balances are achieved. The definitive tibial cut is then performed under navigation guidance, based on this adjusted intraoperative planning. To prevent tibial component loosening due to increased posterior forces, a maximum allowable increase in PTS of 4° relative to the patient's native slope was established, in line with Howell's recommendations [10, 11, 19, 20, 21, 22], to minimise excessive posterior forces on the tibial component.

### Study outcomes

The primary objective of this study was to assess the radiographic accuracy of the NextAR navigation system by comparing the planned coronal and sagittal alignment parameters with those measured postoperatively on standardised weight‐bearing long‐leg radiographs. Radiographic measurements (LDFA, MPTA, PTS and HKA) were performed using TraumaCad digital measurement software (Brainlab AG) on standardised, weight‐bearing long‐leg radiographs by two independent observers (G.C. and D.V.). Each parameter was measured twice, at least 2 weeks apart, and the mean value was used for analysis. Inter‐ and intraobserver reliability were assessed using the intraclass correlation coefficient (ICC), with values > 0.80 considered excellent. For the femoral component, the planned coronal alignment corresponded to the patient's native LDFA. In contrast, the sagittal alignment reflected the optimal flexion of the femoral component determined preoperatively. For the tibial component, the reference parameters were the MPTA and the PTS, as defined intraoperatively following femoral resurfacing and assessment of the surrounding soft tissue.

Furthermore, preoperative and postoperative HKA angles were measured on standardised standing long‐leg radiographs to assess the overall correction of limb alignment. Postoperative measurements were compared with the preoperative 3D plan for parameters determined exclusively during preoperative planning (e.g., LDFA), and with the intraoperatively adjusted plan for parameters potentially modified during surgery based on ligament assessment (e.g., MPTA, PTS and femoral flexion). While not a direct measure of implant placement accuracy, HKA provides a global view of alignment restoration achieved through the AR‐guided strategy.

Secondary outcomes included evaluating short‐term clinical results at a minimum follow‐up of 12 months. Functional outcomes were assessed using two validated patient‐reported outcome measures (PROMs): the Western Ontario and McMaster Universities Osteoarthritis Index (WOMAC) [30] and the Forgotten Joint Score (FJS) [12]. Both questionnaires were administered in their validated Italian versions [28], ensuring appropriate linguistic and cultural adaptation for the study population. Improvements in joint function were further analysed by comparing preoperative and postoperative ROM, specifically evaluating maximum active flexion and extension. The total WOMAC score was used for analysis in this study; individual subdomains such as pain and function were not evaluated separately.

Additionally, the accuracy of preoperative 3D planning in predicting the final size of the femoral and tibial components was evaluated by comparing the planned sizes with the implanted sizes to assess the precision of the NextAR system.

Finally, ROM was measured using a standard handheld goniometer by the operating surgeon during preoperative consultation and at final follow‐up. Measurements were taken with the patient in the supine position, recording maximum active knee flexion and extension to the nearest degree.

### Statistical analysis

Statistical analyses were performed using IBM SPSS Statistics, Version 27.0 (IBM Corp.). Descriptive statistics were reported as mean ± standard deviation (SD) for continuous variables and as frequencies and percentages for categorical variables. A post hoc power analysis was conducted based on the observed accuracy results of this study to estimate the minimum sample size required to statistically confirm that the accuracy of component positioning with the NextAR system exceeds clinically acceptable thresholds. The proportion of cases with an angular error below 2° was 87.8% for the MPTA, 100% for the LDFA, and 82.9% for the PTS. Using a conservative acceptable threshold of 75%, and assuming a significance level of 0.05 with 80% statistical power, the analysis revealed that at least 142 patients would be required to confirm the observed MPTA accuracy, 38 patients for the LDFA, and 372 patients for the PTS. The accuracy of the NextAR system in reproducing planned coronal and sagittal alignment was evaluated using paired *t*‐tests comparing preoperative planning values with postoperative radiographic measurements. The precision of femoral and tibial component size prediction was assessed by calculating the concordance rate between planned and implanted sizes. Improvements in clinical scores were also analysed using paired *t*‐tests. Statistical significance was set at *p* < 0.05.

## RESULTS

### Patient characteristics

A total of 41 patients (41 knees) met the inclusion criteria, completed pre‐ and postoperative radiographic evaluations, and reached a minimum follow‐up of 12 months. The mean age at surgery was 70.9 ± 7.1 years (range, 59.2–79.8), with a predominance of female patients (61%, *n* = 25) (Table 1).

**Table 1:** Patient demographics and baseline clinical characteristics.

Parameter	Value
Number of patients, *N*	41
Age (years), mean ± SD (range)	70.9 ± 7.1 (59.2–79.8)
Female, *n* (%)	25 (61%)
Male, *n* (%)	16 (39%)

Abbreviation: SD, standard deviation.

### Coronal plane alignment

#### Hip‐knee‐ankle angle (HKA)

**Figure 4:**
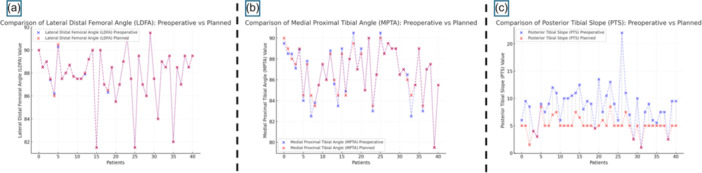
Comparative analysis of preoperative and planned angular measurements. (a) Lateral distal femoral angle (LDFA), (b) medial proximal tibial angle (MPTA) and (c) posterior tibial slope (PTS). Each scatter plot illustrates individual patient data, comparing preoperative (blue) and planned (red) angular values. For LDFA, minimal differences were observed between preoperative and planned measurements, aiming to closely replicate the patient's native femoral anatomy. For MPTA, surgical planning preserved native coronal alignment when tibial wear was absent, with limited variations between conditions. For PTS, a significant reduction was planned across the cohort to facilitate flexion gap closure following posterior cruciate ligament (PCL) excision, as indicated by grey dotted lines connecting preoperative and planned values.

The average preoperative HKA measured on long‐leg standing radiographs was 174.3° ± 3.4° (range: 167.2°–179.8°), indicating a consistent varus alignment (Table 2). The planned postoperative HKA based on CT‐based preoperative planning was 178.1° ± 1.9°, and the actual postoperative HKA measured on radiographs was 177.8° ± 2.1°, resulting in a mean correction of 3.5° from baseline and a deviation of 0.3° from the planned value (*p* < 0.001) (Figure 4d, Table 3).

**Table 2:** Planned parameters, preoperative and intraoperative planning.

Parameter	Preoperative (mean ± SD)	Planned (mean ± SD)
HKA (°)	174.3 ± 3.4	178.1 ± 1.9
LDFA (°)	87.7 ± 2.3	87.7 ± 2.3
MPTA (°)	86.7 ± 2.5	86.8 ± 2.3
PTS (°)	8.5 ± 3.6	5.1 ± 1.6
Femoral flexion (°)	/	3.3 ± 1.6

Abbreviations: HKA, hip‐knee‐ankle angle; LDFA, lateral distal femoral angle; MPTA, medial proximal tibial angle; PTS, posterior tibial slope; SD, standard deviation.

**Table 3:** Accuracy and postoperative radiographic measurements.

Parameter	Postoperative (mean ± SD)	Difference from plan (mean ± SD)	RMSE
HKA (°)	177.8 ± 2.1	0.3 ± 1.5	–
LDFA (°)	87.8 ± 1.9	0.05 ± 0.8	1.15
MPTA (°)	86.7 ± 2.2	0.1 ± 0.6	1.23
PTS (°)	5.3 ± 1.6	0.3 ± 1.3	1.34
Femoral flexion (°)	2.8 ± 1.6	0.5 ± 1.7	1.73

Abbreviations: HKA, hip‐knee‐ankle angle; LDFA, lateral distal femoral angle; MPTA, medial proximal tibial angle; PTS, posterior tibial slope; RMSE, root mean square error; SD, standard deviation.

#### LDFA

**Figure 5:**
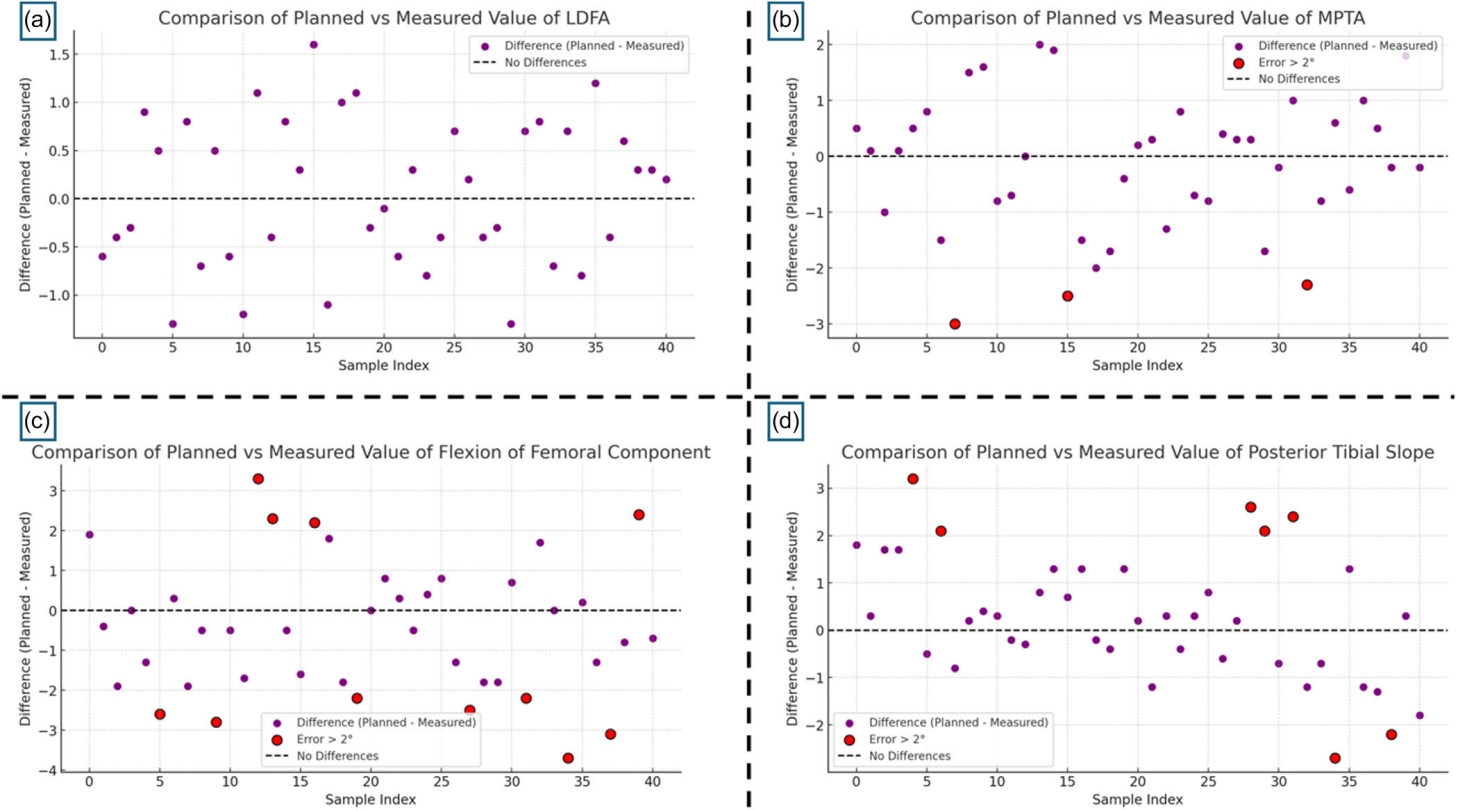
Comparison of planned versus measured angular values. The figure consists of four scatter plots comparing planned and measured values for: (a) lateral distal femoral angle (LDFA), (b) medial proximal tibial angle (MPTA), (c) flexion of the femoral component and (d) posterior tibial slope (PTS). Purple dots represent the difference between planned and measured values. The black dashed line indicates the zero‐difference reference, corresponding to perfect agreement. Red dots highlight cases where the error exceeded 2°.

The preoperative LDFA, measured on standing radiographs, was 87.7° ± 2.3° (range: 82.9°–91.2°), which was maintained during the 3D planning process. The postoperative LDFA measured on radiographs was 87.8° ± 1.9° (range: 83.7°–91.0°), with a mean deviation of −0.1° ± 0.8° (*p* = 0.831) compared to the plan. The root mean square (RMS) error was 1.15°, with errors <0.5° in 14 cases (34.1%), <1° in 26 cases (63.4%) and <2° in 100% of cases (Figure 5a, Table 3). Accordingly, 82.9% of cases (34 out of 41) showed an absolute error in LDFA measurement of less than 1°.

#### MPTA

The preoperative MPTA was 86.7° ± 2.5° (range: 80.9°–90.5°). After intraoperative adjustment based on femoral resurfacing and soft tissue balance, the planned MPTA was 86.8° ± 2.3°, and the postoperative MPTA was 86.7° ± 2.2° (range: 82.5°–90.1°). The difference between the planned and postoperative values was 0.1° ± 0.6° (*p* =  0.841). The RMS error was 1.23°, with <0.5° in 34.1%, <1° in 63.4%, and <2° in 87.8% of patients; five cases (12.2%) showed an error >2° (Figure 5b, Table 3).

### Sagittal plane alignment

#### PTS

The preoperative PTS was 8.5° ± 3.6°. Following intraoperative planning based on soft tissue evaluation, the planned PTS was reduced to 5.1° ± 1.6°, and the postoperative measurement was 5.3° ± 1.6°. The deviation between planned and postoperative PTS was 0.3° ± 1.3° (*p* = 0.573), with an RMS error of 1.34°. Errors were <0.5° in 34.1%, <1° in 53.7% and <2° in 82.9% of cases; seven patients (17%) had deviations >2° (Figure 5d, Table 3).

#### Femoral component flexion

The planned femoral component flexion, determined from preoperative 3D planning to avoid notching, was 3.3° ± 1.6°. The postoperative flexion was 2.8° ± 1.6°, with a mean deviation of 0.5° ± 1.7° (*p* = 0.161). The RMS error was 1.73°, with <0.5° error in 19.6%, <1° in 41.7% and <2° in 73.2% of patients. An error >2° occurred in 11 cases (26.8%) (Figure 5c, Table 3).

### Prediction of component size

The CT‐based preoperative planning demonstrated high accuracy in predicting final implant sizes. The planned femoral component size matched the implanted size in all 41 cases (100%), and the tibial component size was correctly predicted in 39 cases (95.1%), with only a one‐size discrepancy in two patients. While these data reflect the precision of the CT‐based templating process rather than the intraoperative performance of the AR system, they support the reliability of the integrated workflow.

Liner thickness was consistent across patients, with a 10 mm insert used in 92.7% of cases. Although not an endpoint of the study, this consistency supports the overall uniformity of tibial resections and joint balancing achieved through the protocol.

### Clinical outcomes

At the final follow‐up (mean: 14.2 ± 2.3 months), all patients demonstrated statistically significant improvements in both functional scores and ROM (Table 3). The average WOMAC score decreased from 51.5 ± 16.7 (range: 29–86) preoperatively to 13.6 ± 5.3 (range: 6–28) postoperatively (*p* < 0.001). The FJS improved from a preoperative value of 26.2 ± 9.6 (range: 8–41) to 82.2 ± 7.4 (range: 64–97) at final evaluation (*p* < 0.001) (Table 4).

**Table 4:** Clinical outcomes.

Parameter	Preoperative (mean ± SD)	Postoperative (mean ± SD)	*p* value
WOMAC	51.5 ± 16.7	13.6 ± 5.3	<0.001
FJS	26.2 ± 9.6	82.2 ± 7.4	<0.001
ROM, maximum extension (°)	3.3 ± 0.4	0.1 ± 0.3	<0.001
ROM, maximum flexion (°)	103.3 ± 17.4	129.4 ± 7.2	<0.001

Abbreviations: FJS, forgotten joint score; ROM, range of motion; SD; standard deviation; WOMAC, Western Ontario and McMaster Universities Osteoarthritis Index.

In terms of ROM, the average maximum active extension improved from 3.3° ± 0.43° preoperatively to 0.1° ± 0.3° postoperatively (*p* < 0.001), while maximum flexion increased from 103.3° ± 17.4° to 129.4° ± 7.2° (*p* < 0.001), indicating a substantial functional gain following AR‐assisted KA‐TKA (Table 4).

## DISCUSSION

The most relevant finding of this study was the high precision achieved in bone resections using the NextAR AR navigation system combined with a modified KA technique, preserving femoral resurfacing principles while adapting the tibial resection intraoperatively according to ligament balance, as in Howell's method [21, 22], but with the integration of CT‐based planning and AR guidance [6, 27]. In particular, the distal femoral cut showed minimal deviation between planned and postoperative LDFA values (mean difference: 0.05° ± 0.76°), with no outliers beyond 2°. Similarly, differences between planned and measured values were small for femoral component flexion (−0.5° ± 1.7°), MPTA (0.1° ± 0.6°) and PTS (0.3° ± 1.3°), although some outliers were observed (11 [26.8%], 5 [12.2%] and 7 [17%], respectively).

A distinguishing feature of this approach is the delayed intraoperative definition of the tibial cut, performed after femoral resurfacing and guided by real‐time evaluation of ligament balance. This preserves the core principles of calipered KA while introducing a dynamic adjustment of tibial parameters based on functional feedback. Coronal plane changes were minimal, typically limited to cases with bone loss. However, in the sagittal plane, reductions in tibial slope were more frequent and clinically relevant, aimed at managing flexion gap laxity following PCL resection [4, 20].

The CT‐based preoperative planning was also highly predictive in terms of component sizing, with 100% accuracy for femoral components and 95.1% for tibial components. However, it should be noted that implant sizing accuracy primarily reflects the precision of CT‐based planning, rather than the AR navigation system itself, which does not directly influence component selection intraoperatively. This confirms the reliability of the 3D planning process when integrated with AR guidance.

The accuracy observed in restoring the LDFA is particularly notable. The mean deviation from planned alignment was only 0.05° more valgus, with an RMS error of 0.75°. Specifically, for LDFA, no patient exhibited a deviation greater than 2°, and 82.9% remained within 1° of error. This suggests that deviations greater than 1° were confined to a small subset of patients, raising the hypothesis that specific phenotypes—such as those with more pronounced preoperative deformities—may influence the accuracy of execution. Further investigation in larger cohorts is warranted to determine whether specific anatomical patterns contribute to reduced precision. These results are more precise than those reported in recent studies on manually calibrated KA, where RMS errors of approximately 1.4° and rates of <2° deviation around 88% were reported [9, 21, 22, 23]. While calipered techniques have proven accuracy, their reliance on assumptions about cartilage thickness (commonly estimated at 2 mm) may reduce consistency. In contrast, CT‐based planning allows bone‐focused measurements, eliminating this variable and enhancing reproducibility [8, 19].

A key methodological difference between classical KA and the proposed method lies in the timing of tibial cut planning. Here, the MPTA is defined intraoperatively to match the desired tension in the collateral ligaments [1, 37]. The modified KA technique adopted in this study differs from traditional unrestricted KA by integrating an intraoperative evaluation of collateral ligament balance to guide tibial resection, rather than relying solely on restoring native anatomical angles. This approach allows for tailored coronal and sagittal adjustments—particularly of the tibial slope—based on soft tissue behaviour during the procedure, while maintaining the principles of femoral resurfacing typical of KA [1, 6, 27, 37]. In contrast, functional alignment aims to restore limb balance through intraoperative adjustments of both femoral and tibial cuts based on imaging‐derived targets and load sensors, often within defined mechanical ‘safe zones’ [4, 6]. Our technique can therefore be considered a hybrid strategy that maintains the resurfacing principles of KA while incorporating functional alignment principles through real‐time ligament tension assessment and selective tibial modification [1, 6, 27].

This approach may be considered a modified form of KA, where the tibial cut is guided by ligament balance assessment rather than purely replicating the native joint line. Although the preoperative MPTA was preserved in 41.5% of cases, in the remaining 58.5% the tibial alignment was adjusted—albeit modestly in most cases—to optimise varus‐valgus balance. Only six patients required modifications exceeding 2°, suggesting that the system enables fine‐tuning rather than radical changes. While this method aligns closely with the principles of functional alignment (FA), it differs in that no predefined coronal correction thresholds were applied. Moreover, similar to the technique described by Howell, our approach allows for tibial recuts or slope changes when necessary, although recuts were rarely needed in our series [1, 6, 27, 37].

The MCL should maintain a consistent length throughout the ROM but gradually reduce its tension with increasing flexion. This produces a knee that is stable in extension (opening <1 mm) and opens slightly under varus stress in flexion, as previously described [1, 27, 37]. The incorporation of balance evaluation into the workflow drastically reduced the need for tibial recuts—only two were needed in this series, both for a tight but balanced extension gap. None were necessary for coronal malalignment, confirming the technique's effectiveness.

In 41.5% of patients, the preoperative MPTA was preserved, while in 58.5%, the alignment was modified intraoperatively to optimise ligament tension. Of these, 54.2% required a more valgus cut, and 45.8% required a more varus orientation. Most adjustments were modest (0.5°–1°), with only two cases exceeding 2°, both of which were associated with marked preoperative varus deformity and medial tibial bone loss.

In the sagittal plane, similar trends were observed for femoral flexion and PTS. While the precision was slightly lower than in the coronal plane (RMS 1.73 for femoral flexion and 1.34 for PTS), the results were still highly satisfactory. However, the relatively high proportion of cases with deviations exceeding 1° in femoral flexion (58%), PTS (46%) and MPTA (37%) highlights areas where the system's performance is less consistent. These deviations should be critically considered when interpreting the overall accuracy of the AR‐based technique. Nonetheless, despite these variability rates, the overall radiographic precision—particularly in the coronal plane—and the favourable clinical outcomes observed suggest that the system provides reliable guidance for component positioning in the context of modified KA‐TKA.

The ability to preoperatively define femoral component flexion with the navigation system helped avoid notching, a known limitation of KA using short intramedullary guides [7, 10]. In 73.2% of cases, the difference between planned and measured flexion remained below 2°. However, the fact that over a quarter of cases required more than 2° adjustment intraoperatively highlights the limitations of preoperative planning in predicting flexion alignment. It suggests a need for real‐time intraoperative verification and flexibility.

Reduction in PTS was driven by the need to balance the increased flexion gap following PCL resection. Two strategies are available: increasing the posterior condylar offset [17] or reducing the tibial slope. The AR system enabled us to quantify slope reduction and its impact on the flexion gap in real‐time, an advantage over conventional techniques [13, 16, 13, 23, 24, 25].

Beyond precision, the NextAR system offered workflow benefits. With only two infra‐red trackers—mounted on the tibia and femur and reused on different tools during the procedure—the system eliminated the need for external optical arrays. This facilitated uninterrupted communication, minimised assistant interference and improved surgical efficiency [6, 27]. Real‐time intraoperative guidance was provided throughout both femoral and tibial preparation, ensuring consistent accuracy. However, it is worth noting that accurately positioning the tracker can be challenging when pins are inserted freehand. The use of CT‐based planning to guide pin placement may significantly reduce intraoperative struggle and improve system usability.

While several studies have reported high reproducibility and accuracy for RA systems, the evidence remains heterogeneous, and recent systematic reviews have shown conflicting results depending on the specific platform and outcome analysed [2, 3, 9]. Therefore, a more critical appraisal is warranted when interpreting RA performance, particularly given the lack of consistent superiority over conventional or navigated techniques across all domains.

Moreover, it is essential to clarify that the system evaluated in this study does not represent a robotic platform but rather an advanced computer‐assisted navigation system incorporating AR functionalities. The AR component—intended to improve spatial orientation and ergonomics—depends on the use of smart glasses. However, in clinical practice, the use of these devices is not universal; many surgeons have discontinued wearing the headset due to its weight and limited comfort, opting instead to use the system's real‐time feedback via conventional displays. As such, when the smart glasses are not used, the system functions similarly to other modern navigation systems with integrated ligament assessment, as already described in literature more than a decade ago [6, 27].

Clinical outcomes were consistent with the high technical precision achieved. At a minimum follow‐up of 12 months, both PROMs showed significant improvement. The WOMAC score decreased from 51.52 ± 16.7 to 13.6 ± 5.33, and the FJS increased from 26.2 ± 9.6 to 82.2 ± 7.4 (*p* < 0.001 for both), reflecting improvements in function and implant awareness. Objective gains in ROM were also notable, with full extension (from 3.3° ± 0.43° to 0.11° ± 0.28°) and flexion (from 103.3° ± 17.4° to 129.4° ± 7.2°, *p* < 0.001), consistent with the hypothesis that KA contributes to more physiological joint motion [11, 26, 35].

This study has several limitations that should be acknowledged. First, its retrospective design inherently introduces potential biases, including selection bias and confounding factors. Second, the findings are based on a single‐surgeon, single‐centre experience, which may limit their generalisability to broader surgical populations. Third, postoperative alignment was assessed using weight‐bearing long‐leg radiographs, which, although clinically valuable, are less accurate than CT scans for evaluating rotational positioning and PCA alignment. Future studies incorporating postoperative CT imaging are needed to accurately assess rotational errors and provide a more comprehensive evaluation of component positioning. Furthermore, while the study demonstrated excellent short‐term clinical outcomes, the absence of long‐term follow‐up prevents conclusions regarding implant survivorship and durability. Finally, although the novel intraoperative modification of the tibial cut based on ligament balance assessment appears promising, its long‐term effects on joint stability and functional outcomes remain to be determined. Additionally, as in other navigation or robotic platforms, this system still relies on the correct positioning of manual cutting guides, a step that may introduce variability and error. This may partially explain the outliers observed in femoral flexion and PTS accuracy, especially in cases with errors exceeding 2°. Finally, a key limitation of this study is the relatively small sample size, which, despite the encouraging results in terms of accuracy of bone resections and component positioning, does not allow for statistically powered inferential analyses to definitively demonstrate that the observed accuracy exceeds clinically acceptable thresholds, particularly for the MPTA and PTS parameters. Nevertheless, several aspects of the study design contribute to reducing variability, including the single‐surgeon, single‐centre setting, which ensures consistency in surgical technique, and the strict selection criteria with standardised radiographic and clinical evaluations. Considering these factors, the results should be interpreted as preliminary and descriptive, providing promising insights that align with existing literature on AR‐assisted navigation and KA in TKA. This work should be considered a pilot study, offering preliminary data that suggest the NextAR system may assist in achieving accurate component positioning and serve as a basis for the design of future prospective or multicenter trials with adequate statistical power to confirm these findings. Moreover, the absence of a control or comparator group (e.g., conventional instrumentation or standard navigation) limits the ability to attribute the observed outcomes specifically to the AR system.

## CONCLUSION

This study demonstrates that AR‐assisted KA‐TKA using the NextAR system enables the precise execution of femoral and tibial bone cuts, particularly in restoring coronal alignment, such as the LDFA, with variable results in sagittal parameters, including femoral flexion and PTS. The integration of real‐time soft tissue tension assessment enabled intraoperative adjustments to optimise ligament balance while respecting the principles of KA. Preoperative CT‐based planning showed high accuracy in predicting femoral and tibial component sizes, and the need for tibial recuts was limited. Clinically significant improvements were observed in patient‐reported outcomes (WOMAC and FJS) and joint ROM at short‐term follow‐up. However, the observed rates of deviation greater than 1° in MPTA, PTS, and femoral flexion suggest that precision may vary by parameter and warrant further investigation. These findings, although encouraging, should be interpreted in consideration of the study's limitations, including its retrospective design, single‐surgeon setting and the absence of a control group. Further prospective multicenter studies with long‐term follow‐up are needed to confirm these results and determine their impact on implant durability and functional performance.

## AUTHOR CONTRIBUTIONS

Giorgio Cacciola and Francesco Bosco contributed to the conceptualisation and design of the study. Daniele Vezza and Gianpaolo Gazziero were responsible for data collection. Luigi Sabatini supervised the project. Giorgio Cacciola, Francesco Carturan and Marco Bufalo contributed to data interpretation and manuscript preparation. All authors reviewed and approved the final version of the manuscript.

## CONFLICT OF INTEREST STATEMENT

Luigi Sabatini is a paid consultant for Medacta International. The remaining authors declare no conflict of interest. Although Medacta International covered the article processing charges, the company had no role in the scientific conduct of the study.

## ETHICS STATEMENT

This study was approved by the Territorial Ethics Committee A.O.U. Città della Salute e della Scienza di Torino (approval number 123/2024). All patients provided informed consent for the use of their clinical data for research and publication purposes, in accordance with institutional and ethical standards.

## Data Availability

The data that support the findings of this study are available on request from the corresponding author. The data are not publicly available due to privacy or ethical restrictions.

## References

[1] Ascani D , Mazzà C , De Lollis A , Bernardoni M , Viceconti M . A procedure to estimate the origins and the insertions of the knee ligaments from computed tomography images. J Biomech. 2015;48(2):233–237. 25512017 10.1016/j.jbiomech.2014.11.041

[2] Bennett KM , Griffith A , Sasanelli F , Park I , Talbot S . Augmented reality navigation can achieve accurate coronal component alignment during total knee arthroplasty. Cureus. 2023;15(2):e34607. 36883097 10.7759/cureus.34607 PMC9985958

[3] Cacciola G , Bosco F , Vezza D , Carturan F , De Meo F , Cavaliere P , et al. Augmented reality in knee arthroplasty: a scoping review of the current evidence. AME Surg J. 2025;5:2.

[4] Cacciola G , Giustra F , Bosco F , Vezza D , Pirato F , Braconi L , et al. No significant clinical differences between native or reduced posterior tibial slope in kinematically aligned total knee replacement with posterior cruciate‐retaining. J Orthop. 2024;54:32–37. 38524363 10.1016/j.jor.2024.03.023 PMC10957378

[5] Daniel C , Ramos O . Augmented reality for assistance of total knee replacement. J Electr Comput Eng. 2016;2016:9358369.

[6] Fucentese SF , Koch PP . A novel augmented reality‐based surgical guidance system for total knee arthroplasty. Arch Orthop Trauma Surg. 2021;141(12):2227–2233. 34698930 10.1007/s00402-021-04204-4 PMC8595230

[7] Gangadharan R , Deehan DJ , McCaskie AW . Distal femoral resection at knee replacement—The effect of varying entry point and rotation on prosthesis position. Knee. 2010;17(5):345–349. 19875296 10.1016/j.knee.2009.09.011

[8] Giurazza G , Campi S , Hirschmann MT , Franceschetti E , Tanzilli A , Gregori P , et al. Cartilage thickness can be accurately measured intraoperatively in total knee arthroplasty: a step further in calipered kinematic alignment. J Exp Orthop. 2025;12(1):e70155. 39867675 10.1002/jeo2.70155 PMC11763056

[9] Giurazza G , Caria C , Campi S , Franceschetti E , Papalia GF , Basciani S , et al. Femoral cartilage thickness measured on MRI varies among individuals: time to deepen one of the principles of kinematic alignment in total knee arthroplasty. A systematic review. Knee Surg Sports Traumatol Arthrosc. 2025;33(2):634–645. 39135541 10.1002/ksa.12408

[10] Howell SM , Akhtar M , Nedopil AJ , Hull ML . Reoperation, implant survival, and clinical outcome after kinematically aligned total knee arthroplasty: a concise clinical follow‐up at 16 years. J Arthroplasty. 2024;39(3):695–700. 37659680 10.1016/j.arth.2023.08.080

[11] Howell SM , Shelton TJ , Hull ML . Implant survival and function ten years after kinematically aligned total knee arthroplasty. J Arthroplasty. 2018;33(12):3678–3684. 30122435 10.1016/j.arth.2018.07.020

[12] Howell SM , Zabiba A , Nedopil AJ , Hull ML . The forgotten joint score after total knee arthroplasty with a kinematic alignment‐optimized femoral component matches total hip arthroplasty. Knee Surg Sports Traumatol Arthrosc. 2025;33(10):3646–3653. 40450564 10.1002/ksa.12712 PMC12459319

[13] Kayani B , Konan S , Horriat S , Ibrahim MS , Haddad FS . Posterior cruciate ligament resection in total knee arthroplasty: the effect on flexion‐extension gaps, mediolateral laxity, and fixed flexion deformity. Bone Jt J. 2019;101–B(10):1230–1237. 10.1302/0301-620X.101B10.BJJ-2018-1428.R2 31564152

[14] Lee BS , Cho HI , Bin SI , Kim JM , Jo BK . Femoral component varus malposition is associated with tibial aseptic loosening after TKA. Clin Orthop Rel Res. 2018;476(2):400–407. 10.1007/s11999.0000000000000012 PMC6259714 29389790

[15] Lex JR , Koucheki R , Toor J , Backstein DJ . Clinical applications of augmented reality in orthopaedic surgery: a comprehensive narrative review. Int Orthop. 2023;47(2):375–391. 35852653 10.1007/s00264-022-05507-w

[16] Li C , Li T , Zhang Z , Huang H , Rong C , Zhu W , et al. Robotic‐arm assisted versus conventional technique for total knee arthroplasty: early results of a prospective single centre study. Int Orthop. 2022;46(6):1331–1338. 35224668 10.1007/s00264-022-05351-y

[17] Malavolta M , Compagnoni R , Mezzari S , Calanna F , Pastrone A , Randelli P . Good clinical results using a modified kinematic alignment technique with a cruciate sacrificing medially stabilised total knee arthroplasty. Knee Surg Sports Traumatol Arthrosc. 2022;30(2):500–506. 32748231 10.1007/s00167-020-06196-x

[18] Mancino F , Cacciola G , Malahias MA , De Filippis R , De Marco D , Di Matteo V , et al. What are the benefits of robotic‐assisted total knee arthroplasty over conventional manual total knee arthroplasty? A systematic review of comparative studies. Orthop Rev. 2020;12(Suppl 1):8657. 10.4081/or.2020.8657 PMC7459388 32913593

[19] Nam D , Lin KM , Howell SM , Hull ML . Femoral bone and cartilage wear is predictable at 0° and 90° in the osteoarthritic knee treated with total knee arthroplasty. Knee Surg Sports Traumatol Arthrosc. 2014;22(12):2975–2981. 24839078 10.1007/s00167-014-3080-8

[20] Nedopil AJ , Delman C , Howell SM , Hull ML . Restoring the patient's pre‐arthritic posterior slope is the correct target for maximizing internal tibial rotation when implanting a PCL retaining TKA with calipered kinematic alignment. J Pers Med. 2021;11(6):516. 34200031 10.3390/jpm11060516 PMC8228254

[21] Nedopil AJ , Dhaliwal A , Howell SM , Hull ML . A surgeon that switched to unrestricted kinematic alignment with manual instruments has a short learning curve and comparable resection accuracy and outcomes to those of an experienced surgeon. J Pers Med. 2022;12(7):1152. 35887649 10.3390/jpm12071152 PMC9320158

[22] Nedopil AJ , Howell SM , Hull ML . Deviations in femoral joint lines using calipered kinematically aligned TKA from virtually planned joint lines are small and do not affect clinical outcomes. Knee Surg Sports Traumatol Arthrosc. 2020;28(10):3118–3127. 31768572 10.1007/s00167-019-05776-w

[23] Oka S , Matsumoto T , Muratsu H , Kubo S , Matsushita T , Ishida K , et al. The influence of the tibial slope on intra‐operative soft tissue balance in cruciate‐retaining and posterior‐stabilized total knee arthroplasty. Knee Surg Sports Traumatol Arthrosc. 2014;22(8):1812–1818. 23689963 10.1007/s00167-013-2535-7

[24] Okazaki K , Tashiro Y , Mizu‐uchi H , Hamai S , Doi T , Iwamoto Y . Influence of the posterior tibial slope on the flexion gap in total knee arthroplasty. Knee. 2014;21(4):806–809. 24856090 10.1016/j.knee.2014.02.019

[25] Oshima Y , Majima T , Iizawa N , Hoshikawa N , Takahashi K , Takai S . The Influence of posterior cruciate ligament resection on tibiofemoral joint gap in varus osteoarthritic knees. J Knee Surg. 2022;35(3):323–330. 32659819 10.1055/s-0040-1713810

[26] Risitano S , Cacciola G , Sabatini L , Capella M , Bosco F , Giustra F , et al. Restricted kinematic alignment in primary total knee arthroplasty: a systematic review of radiographic and clinical data. J Orthop. 2022;33:37–43. 35812351 10.1016/j.jor.2022.06.014 PMC9263746

[27] Sabatini L , Ascani D , Vezza D , Massè A , Cacciola G . Novel surgical technique for total knee arthroplasty integrating kinematic alignment and real‐time elongation of the ligaments using the NextAR system. J Pers Med. 2024;14(8):794. 39201986 10.3390/jpm14080794 PMC11355594

[28] Sansone V , Fennema P , Applefield RC , Marchina S , Ronco R , Pascale W , et al. Translation, cross‐cultural adaptation, and validation of the Italian language forgotten joint score‐12 (FJS‐12) as an outcome measure for total knee arthroplasty in an Italian population. BMC Musculoskelet Disord. 2020;21(1):23. 31926561 10.1186/s12891-019-2985-2 PMC6955087

[29] Smith PN , Gill DR , McAuliffe MJ , McDougall C , Stoney JD , Vertullo CJ , et al. Hip, knee and shoulder arthroplasty: 2023 annual report. Australian Orthopaedic Association National Joint Replacement Registry, AOA: Adelaide, South Australia;2023.

[30] Spece H , Kurtz MA , Piuzzi NS , Kurtz SM . Patient‐reported outcome measures offer little additional value two years after arthroplasty: a systematic review and meta‐analysis. Bone Jt J. 2025;107–B(3):296–307. 10.1302/0301-620X.107B3.BJJ-2024-0910.R1 40025985

[31] Teeter MG , Naudie DD , McCalden RW , Yuan X , Holdsworth DW , MacDonald SJ , et al. Varus tibial alignment is associated with greater tibial baseplate migration at 10 years following total knee arthroplasty. Knee Surg Sports Traumatol Arthrosc. 2018;26(6):1610–1617. 29147742 10.1007/s00167-017-4765-6

[32] Tsukada S , Kizaki K , Saito M , Kurosaka K , Hirasawa N , Ogawa H . Femoral prosthesis alignment of augmented reality‐assisted versus accelerometer‐based navigation in total knee arthroplasty: a noninferiority analysis. J Orthop Sci. 2024;29(6):1417–1422. 37925295 10.1016/j.jos.2023.10.011

[33] Tsukada S , Ogawa H , Nishino M , Kurosaka K , Hirasawa N . Augmented reality‐based navigation system applied to tibial bone resection in total knee arthroplasty. J Exp Orthop. 2019;6(1):44. 31712907 10.1186/s40634-019-0212-6 PMC6848533

[34] Tsukada S , Ogawa H , Nishino M , Kurosaka K , Hirasawa N . Augmented reality‐assisted femoral bone resection in total knee arthroplasty. JB JS Open Access. 2021;6(3):e21.00001. 10.2106/JBJS.OA.21.00001 PMC8301282 34316529

[35] Van Essen J , Stevens J , Dowsey MM , Choong PF , Babazadeh S . Kinematic alignment results in clinically similar outcomes to mechanical alignment: Systematic review and meta‐analysis. Knee. 2023;40:24–41. 36403396 10.1016/j.knee.2022.11.001

[36] Verhey JT , Haglin JM , Verhey EM , Hartigan DE . Virtual, augmented, and mixed reality applications in orthopedic surgery. Int J Med Robot. 2020;16(2):e2067. 31867864 10.1002/rcs.2067

[37] Viceconti M , Ascani D , Mazzà C . Pre‐operative prediction of soft tissue balancing in knee arthoplasty part 1: effect of surgical parameters during level walking. J Orthop Res. 2019;37(7):1537–1545. 30908694 10.1002/jor.24289 PMC6617758

[38] Wang L , Sun Z , Zhang X , Sun Z , Wang J . A hololens based augmented reality navigation system for minimally invasive total knee arthroplasty. Lect Notes Comput Sci. 2019;11745:519–530.

[39] Zhang J , Ndou WS , Ng N , Gaston P , Simpson PM , Macpherson GJ , et al. Robotic‐arm assisted total knee arthroplasty is associated with improved accuracy and patient reported outcomes: a systematic review and meta‐analysis. Knee Surg Sports Traumatol Arthrosc. 2022;30(8):2677–2695. 33547914 10.1007/s00167-021-06464-4 PMC9309123

